# Perforator flaps in the hand

**DOI:** 10.1186/1753-6561-9-S3-A54

**Published:** 2015-05-19

**Authors:** Fortune Iwuagwu

**Affiliations:** 1 St Andrew’s Centre for Plastic Surgery, Broomfield Hospital, Essex, CM1 7ET, UK

## Introduction

The need for an appropriate flap cover to protect vital structures, restore and preserve function and aesthetics of the hand following tissue loss remains a challenge to the reconstructive hand surgeon. Following the description of the angiosome concept by Taylor and Palmer and the pioneering work of authors such as Koshima & Soeda, Kroll, and Rosenfield, in the late 1980s, the perforator flap technique has moved to the centre stage in reconstructive flap surgery.

## Materials and methods

A literature review of the ‘named’ perforator flaps which have been used in the hand and published in peer reviewed English literature using keywords in the title and abstract (perforator flap, hand, palm, dorsum of hand, digits fingers) in MEDLINE served as the framework for this presentation. A further review of the references in the core articles for other perforator based flaps was also done. Clinical examples of the indications, pedicle peculiarities, ideal recipient sites and specific donor site problems of some of the workhorse perforator flaps from my personal experience are also presented.

## Results

There were fifty core articles with ‘named’ perforator flaps. Despite the attempts to achieve a standardized nomenclature for these flaps (Blondeel 2002, 2003), it is clear that the terminology is lacking in universal applicability or acceptance making communication and comparison of literature on ‘perforator’ flaps difficult.

Pre-operative planning involves mostly a good knowledge of the anatomy and the use of the Doppler probe and occasionally imaging investigations such as duplex ultrasound, MRI and other modalities. Once the perforator is identified, the flap design and movement is based on traditional principles of plastic surgery such as rotation, transposition, V-Y flaps, pedicled, free, ad hoc or freestyle. But the conventional flap design rules of length and width ratios do not apply (Lee et al 2009).

The ‘named’ perforator flaps can be grouped into local (harvested from the hand itself), regional (from the upper limb excluding the hand) and distant (from the rest of the body); in other words, based on the distance of the donor site from the recipient site (the hand being the reference point). Examples (with glabrous and non glabrous subgroups) are as follows:

## Local

### Glabrous

Reverse thenar perforator, (Seyhan 2009) reverse palmar perforator flap, palmar perforator (Omokawa 2001, 2002; Kim and Hwang 2005), superficial palmar branch of radial artery flap (SUPBRA) (Iwuagwu et al 2013), ulnar palmar digital artery perforator (Uchida et al 2009), digital artery perforator flaps (Koshima et al 2006, Basat et al 2013) and palmar intermetacarpal perforator flap (Pellissier et al 2009).

### Non-glabrous

Dorsal hand or metacarpal artery perforator flap (Quaba and Davison 1990), dorsal digital artery perforator flap (Kawatsu and Ishikawa 2009) and V-Y flaps based on the perforator concept (Iwuagwu and Misra 2007).

## Regional (all non glabrous)

Distal forearm perforator flap (radial artery perforators) (Tancharoen et al 2013); distal Ulnar artery perforator flap (Unal et al 2011; Inada et al 2004.); proximal ulnar artery perforator (Xiao et al 2013); the dorsoradial forearm perforator flap based on perforators either from the posterior interesseous artery or interesseous recurrent artery or the common interesseous artery or the descending branch of the radial recurrent artery (Gao et al 2011).

## Distant

### Non-glabrous

**Anterior trunk** -- Pectoral intercostal cutaneous perforator flap (Oki et al 2009), deep inferior epigastric perforator flap (DIEP)(Shang et al 2011, Chew et al 2008), posterior trunk - circumflex scapular artery perforator flap (Brandford et al 2009), lattismus dorsal artery perforator flap (Lin and Chen 2014).

**Thighs/groin** – (anterolateral thigh flap (ALT)(Misani et al 2013, Namazi 2014). lateral circumflex femoral artery perforator flap (LCFA)(Hocaoglu et al 2013), superficial circumflex iliac artery perforator flap (Koshima et al 2004).

**Lower leg** -- posterior tibial artery perforator flap (PTP) Zhao et al 2011; medial sural artery perforator flap (SAP) (Lin et al 2011), soleus perforator flap and peroneal perforator flap (Kawamura et al 2005).

### Glabrous

Foot - medialis pedis perforator (Uygur et al 2007), medial plantar artery perforator flaps (Lai et al 2010).

Donor site closure ranged from direct closure to skin grafting and occasionally closure by means of another flap.

There were very contrasting views of donor site morbidity with most authors subjectively judging the morbidity of the donor site for their chosen flap to be minor or acceptable as opposed to the donor sites of other flaps.

## Discussion

The increased appreciation of the perforator concept in reconstructive hand surgery has enabled surgeons to harvest flaps of sufficient size or ‘tailor made size’ that are much thinner and pliable than would have been feasible with reduced donor site morbidity and very importantly avoiding the sacrifice of major blood vessels. A grater precision of insetting of the flaps is possible, further reducing the need for secondary procedures.

For regional and distant perforator flaps, the ability to isolate and define the ‘final pathway’ of blood supply to the skin has enabled accurate placement of expanders and recruitment of more skin safely with less donor site morbidity (Hocaoglu et al 2013), safe and radical thinning of flaps making them more pliable Gao et al 1994, Oki et al 2009, Liu et al 2010), intra adipose dissection of the perforator with recruitment of greater length of pedicle (Kimura et al 2008) and lends well to raising multiple perforator flaps or composite flaps for correction of different tissue defects in one stage (Meky and Safoury 2013).

The local perforator flaps have added advantage that they provide ‘like for like’ tissue in colour, texture and resilience, one site operation, early and one site rehabilitation of the hand but suffer from limited availability.

Excellent microsurgical skills are necessary for surgery of free perforator flaps especially the smaller ones utilised on the digits.

## Conclusion

As the functional donor site morbidity has been reduced by this technique, hand surgeons have to be more conscious of the aesthetic morbidity of using certain donor sites in the long term. The versatility and the gains with this concept have made perforator flaps a welcome addition to the reconstructive armory of the hand surgeon.

